# LRP1 plays a major role in the amyloid-β clearance in microglia

**DOI:** 10.1186/1750-1326-8-S1-P33

**Published:** 2013-09-13

**Authors:** Aurelie N’Songo, Takahisa Kanekiyo, Guojun Bu

**Affiliations:** 1 Department of Neuroscience, Mayo Clinic, Jacksonville Fl, USA

## Background

Alzheimer’s disease (AD), a progressive neurodegenerative disorder and the most prevalent type of dementia in the elderly, is characterized by the accumulation and deposition of amyloid-β (Aβ) peptides and hyperphosphorylated tau in the brain. Impairment of Aβ metabolism induces the formation of toxic Aβ oligomers as well as the deposition of Aβ in intraneuronal spaces and senile plaques, ultimately resulting in neuronal death. While familial AD is known to be caused by genetic mutations leading to an increase in Aβ production, several lines of evidence suggest that sporadic AD is due to an impairment of Aβ clearance. Aβ is cleared from the central nervous system by elimination through the blood-brain barrier, extracellular proteolytic degradation or cellular uptake and subsequent lysosomal degradation. The low-density lipoprotein receptor-related protein 1 (LRP1) has been shown to play a major role in Aβ metabolism in neurons, astrocytes and brain vessels. LRP1 is a large transmembrane receptor which mediates endocytosis of more than 30 ligands including apolipoprotein E and αt2-macroglobulin. Microglia cells are the resident immune and phagocytic cells in the brain and are likely involved in the pathogenesis of AD by contributing to Aβ clearance. Thus, we focused on roles of LRP1 in Aβ clearance in microglia.

## Materials and methods

Mouse microglial BV2 cells and primary microglia from wild type C57BL/6 mice were used in this study. Knockdown of LRP1 was performed by transfection with LRP1-specific siRNA using Lipofectamine 2000 (Invitrogen), and cells were used for analysis 48 hours after transfection. Control and LRP1-suppressed cells were incubated with fluorescently labeled Aβ42 or microspheres, which are internalized through phagocytosis, and then cellular uptake of these molecules was quantified by FACS after 4 hours of incubation. Furthermore, the cellular localization of fluorescently labeled Aβ42 was assessed using confocal laser microscopy.

## Results

LRP1 is highly expressed in both BV2 cells and primary mouse microglia cells. While microglial cells efficiently internalized Aβ, LRP1-suppressed cells showed a decrease of Aβ42 uptake when analyzed by FACS. Consistent with FACS results, we observed less internalized Aβ in LRP1-suppressed microglia cells detected primarily in the lysosomal compartments by confocal microscopy after incubation with Aβ compared to control cells. These results indicate that internalized Aβ is targeted for lysosomal trafficking in the microglia. We also found that the uptake of microspheres was suppressed by the deletion of LRP1 in microglia, suggesting that LRP1 mediates Aβ phagocytosis and subsequent degradation in microglia.

## Conclusion

Our results indicate that LRP1 plays an important role in cellular uptake of Aβ in microglia. The disturbances of LRP1-mediated Aβ clearance in microglia might be involved in AD pathogenesis.

